# Non-human Primate Schlafen11 Inhibits Production of Both Host and Viral Proteins

**DOI:** 10.1371/journal.ppat.1006066

**Published:** 2016-12-27

**Authors:** Alex C. Stabell, John Hawkins, Manqing Li, Xia Gao, Michael David, William H. Press, Sara L. Sawyer

**Affiliations:** 1 BioFrontiers Institute, University of Colorado Boulder, Boulder, CO, United States of America; 2 Institute for Computational Engineering and Sciences, University of Texas at Austin, TX, United States of America; 3 Division of Biological Sciences, UCSD Moores Cancer Center, University of California San Diego, La Jolla, CA, United States of America; 4 Department of Integrative Biology, University of Texas at Austin, Austin, TX, United States of America; University of North Carolina at Chapel Hill, UNITED STATES

## Abstract

Schlafen11 (encoded by the *SLFN11* gene) has been shown to inhibit the accumulation of HIV-1 proteins. We show that the *SLFN11* gene is under positive selection in simian primates and is species-specific in its activity against HIV-1. The activity of human Schlafen11 is relatively weak compared to that of some other primate versions of this protein, with the versions encoded by chimpanzee, orangutan, gibbon, and marmoset being particularly potent inhibitors of HIV-1 protein production. Interestingly, we find that Schlafen11 is functional in the absence of infection and reduces protein production from certain non-viral (GFP) and even host (Vinculin and GAPDH) transcripts. This suggests that Schlafen11 may just generally block protein production from non-codon optimized transcripts. Because Schlafen11 is an interferon-stimulated gene with a broad ability to inhibit protein production from many host and viral transcripts, its role may be to create a general antiviral state in the cell. Interestingly, the strong inhibitors such as marmoset Schlafen11 consistently block protein production better than weak primate Schlafen11 proteins, regardless of the virus or host target being analyzed. Further, we show that the residues to which species-specific differences in Schlafen11 potency map are distinct from residues that have been targeted by positive selection. We speculate that the positive selection of *SLFN11* could have been driven by a number of different factors, including interaction with one or more viral antagonists that have yet to be identified.

## Author Summary

Schlafen11 was recently identified as a human antiviral protein with activity against HIV-1. Here we show that some nonhuman primate versions of Schlafen11 are much stronger at blocking the accumulation of viral proteins than is human Schlafen11. These relatively larger phenotypes of nonhuman primate Schlafen11 allowed us to explore further into the mechanism of this protein. We present data showing that Schlafen11 may not be a classic restriction factor, but rather an interferon-stimulated gene with broad ability to inhibit protein production from many host and viral transcripts, creating a general antiviral state in the cell.

## Introduction

RNA viruses pose a major threat to public health because of their potential to rapidly adapt to the human host after transmission from animal reservoirs [1]. In general, the genetic barriers to cross-species transmission of viruses are poorly understood. A notable exception exists in the case of retroviruses, where a number of well-characterized restriction factors have been shown to potently block viral replication in non-native hosts [2]. Restriction factors recognize viruses and directly interfere with, or restrict, viral lifecycles. Restriction factors have been identified to act at several different stages of the retroviral lifecycle including uncoating (TRIM5α), reverse transcription (APOBEC3s, SAMHD1), import/integration (MxB), and budding (Tetherin) [3–11]. These restriction factors all recognize virus-associated molecular patterns, such as the viral capsid or single stranded DNA exposed during reverse transcription. Zoonotic transmission of retroviruses is rare, only occurring when a virus is able to adapt to subvert manifold human innate immune defenses simultaneously. Most notably, simian immunodeficiency viruses (SIVs) have on several occasions adapted to humans, becoming the human immunodeficiency viruses HIV-1 and HIV-2 [12–14].

Recently, Schlafen11 (encoded by the *SLFN11* gene) was shown to restrict HIV-1 replication at the step of protein translation [15]. Interestingly, Schlafen11 was reported to block translation of viral but not host proteins. This was shown to be related to the different patterns of codon usage observed between host and retroviral transcripts. It has long been known that many RNA viruses do not have the same codon bias as their hosts [16]. Reasons proposed for this differential codon usage include constraints imposed by RNA secondary-structure, mutational biases of viral polymerases, and translational rate requirements for proper protein folding [17–19]. Evidence exists to support each of these hypotheses, and all evidence is in line with non-optimal codon usage being generally beneficial for RNA viruses [20]. Thus, the discovery of Schlafen11 yet again revealed the amazing power of the immune system to take advantage of any possible difference between self and non-self as a way to target and destroy viruses.

Many primate restriction factor genes with activity against HIV-1 have been shown to be subject to evolutionary “arms race” dynamics [14,21–33]. Arms races can occur when a direct interaction exists between a host-encoded protein and a virus-encoded protein. For instance, host restriction factors interact with viral proteins that are either the target of restriction (e.g. TRIM5α and HIV-1 capsid [34]) or antagonist proteins that the virus uses to block restriction (e.g. APOBEC3G and HIV-1 Vif [35]). In these instances, both genomes experience constant selection for new allelic protein variants that weaken or strengthen this physical interaction, depending on which is beneficial to each party [36,37]. This creates a situation of runaway evolution, where both sides must continually adapt to keep step with the other. Arms races may play out over millions of years of evolution, during which many speciation events may occur. This process leads to restriction factors becoming species-specific, which can be studied in the laboratory to enhance our understanding of zoonosis and disease emergence. When a cross-species viral transmission event occurs, the virus must adapt in order to subvert the restriction factors as they exist in the new host, or perish.

In this study, we show that the *SLFN11* gene is evolving under positive selection in simian primates and has become species-specific in its activity against HIV-1. The activity of human Schlafen11 is weak compared to that of some other primate versions of this protein, with the Schlafen11 proteins encoded by chimpanzee, orangutan, gibbon, and marmoset being particularly potent inhibitors of HIV-1 protein production. Interestingly, the strong inhibitors such as marmoset Schlafen11 consistently block protein production better than weak primate Schlafen11 proteins, regardless of the virus being analyzed. Further, we show that the ability of Schlafen11 to decrease protein levels is independent of viral infection altogether: Schlafen11 reduces protein production of viral, non-viral (GFP), and even host proteins (in particular if they are poorly codon optimized), even outside of the context of any infection. Because the activity of each primate Schlafen11 is consistent across the target proteins tested, species-specific differences in Schlafen11 appear to have more to do with differences in the basic function of these homologs rather than their ability to recognize particular viral patterns. In line with this model, we map the residues responsible for species-specific differences in Schlafen11 and find that they are distinct from residues that have been targeted by positive selection. Therefore, because Schlafen11 inhibits protein accumulation from diverse target genes and is upregulated by interferon [38], it may be better thought of as a classic, antiviral interferon-stimulated gene than a restriction factor that is specific to a given virus or viral family. The positive selection in the *SLFN11* gene may have been caused by selective pressure exerted by one or more unidentified antagonists encoded by retroviruses or other viruses, past or present. Why different primate versions of Schlafen11 have such variable potencies at inhibiting protein production remains unknown, but we can speculate that there may be a cost associated with Schlafen11 activity, and that therefore it has been selected for reduced potency in some species.

## Results

### The *SLFN11* gene is evolving under recurrent positive selection in primates

**Fig 1:**
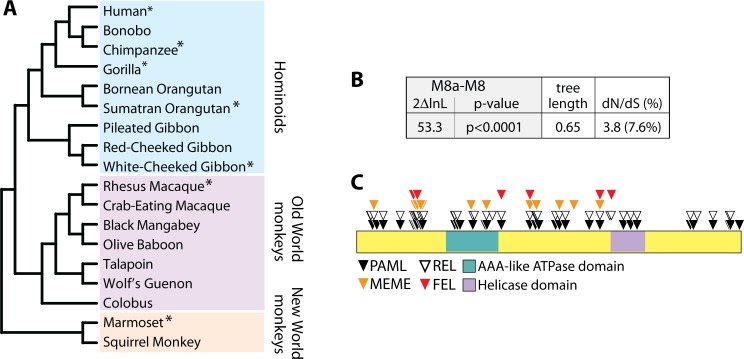
The *SLFN11* gene has evolved under positive selection in primates. **(A)** A phylogeny of the primate *SLFN11* gene sequences used in this analysis. Sequences obtained from online databases are indicated with asterisks, the others were generated in this study. **(B)** Table summarizing the likelihood ratio test between the M8 and M8a models in PAML. The 2ΔlnL value (twice the difference in the natural log of the likelihoods) for M8 versus M8a is shown, along with the p-value with which the neutral model M8a is rejected in favor of the model of positive selection. The tree length of the *SLFN11* gene alignment was 0.65, and 7.6% of codons were assigned a dN/dS = 3.8. PAML analysis was repeated using different codon models (f61, 3x4) and different ω_0_ seed values, and in all cases results converged. **(C)** Residues corresponding to codons with dN/dS > 1 are indicated on a schematic of the Schlafen11 protein.

To investigate the evolutionary history of the *SLFN11* gene in simian primates, we aligned *SLFN11* orthologs from 18 species, representing approximately 40 million years of primate evolution (Fig 1A). Seven of these sequences were obtained from primate genome projects (asterisks in Fig 1A). The remaining 11 were directly sequenced from cDNA libraries generated using extracted RNA from primate cell lines. We then analyzed this *SLFN11* multiple alignment for codon positions enriched for nonsynonymous substitutions relative to synonymous substitutions (dN/dS>1), indicative of positive, or diversifying, selection in favor of amino-acid altering mutations. Using four common tests, we found strong evidence for positive selection in the *SLFN11* gene (S1 Table). For instance, using PAML we found that a model of neutral evolution (M8a) was rejected in favor of a model of positive selection (M8; p < 0.0001). In the model of positive selection, many (7.6%) of the codon positions in this gene were assigned a value of dN/dS > 1 (Fig 1B). Codons identified to be evolving under positive selection by each of the four tests are listed in S1 Table, and correspond to residues spread throughout the length of the protein (Fig 1C). These findings suggest the *SLFN11* gene has encountered one or multiple pressures that have driven selection in favor of protein-altering mutations in this gene.

### Schlafen11 blocks protein production by retroviruses in a species-specific fashion

**Fig 2:**
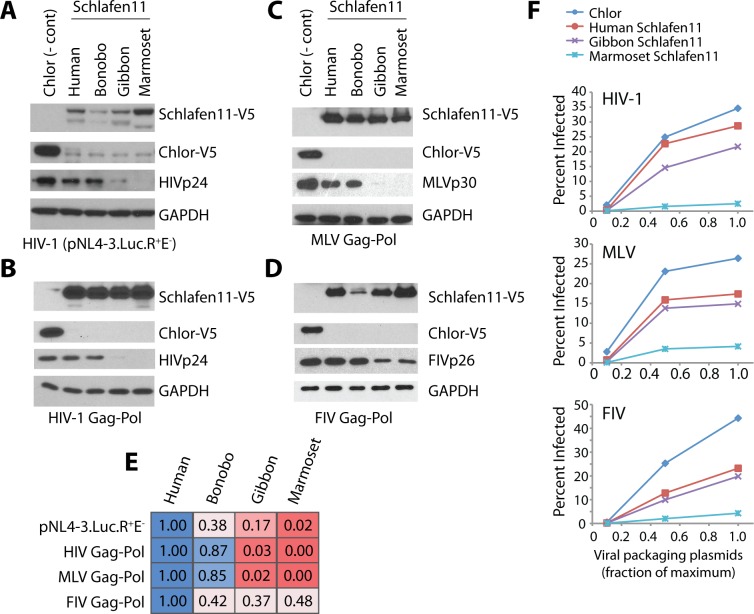
Primate versions of Schlafen11 differentially inhibit retroviral protein production. Plasmids encoding Schlafen11-V5 from the indicated primate species, or a V5-tagged chloramphenichol acetyltransferase (Chlor) gene as a negative control, were co-transfected into 293T cells along with plasmids encoding **(A)** a nearly full-length HIV clone (pNL4-3.Luc.R^+^E^-^), **(B)** HIV-1 Gag-Pol-RRE and HIV-1 Rev, **(C)** MLV Gag-Pol, **(D)** FIV Gag-Pol. Immunoblotting was used to monitor protein production of chloramphenichol acetyltransferase (Chlor-V5), Schlafen11-V5, GAPDH, and viral proteins. Panels A-D are representative blots from 3 or more experimental replicates. **(E)** Bands from panels A-D were quantified to show the relative effect of each Schlafen11 homolog. Each experiment was normalized to human Schlafen11. As increasing Schlafen11 activity as seen, the color of the box changes linearly along the blue to red color spectrum (see materials and methods for quantification procedure). **(F)** Viruses were packaged in 293T cells in the presence or absence of Schlafen11. Increasing amounts of the plasmids necessary for virus packaging were co-transfected along with a constant amount of plasmid expressing human, gibbon, or marmoset Schlafen11 (or Chlor as a negative control). The resulting virions were then used to infect 2.5x10^5^ 293T cells and the percentage of infected cells was scored by flow cytometry (GFP+). These data are representative of two independent experiments. Herein, “Chlor” is used as an abbreviation instead of the standard “CAT,” since the latter is also the name of another mammal and could therefore cause confusion.

We next investigated whether the evolutionary sequence divergence that has occurred in the *SLFN11* gene results in species-specific effects on HIV-1. We cloned into a mammalian expression plasmid *SLFN11* from human and three simian primate species (bonobo, *Pan paniscus*; red-cheeked gibbon, *Nomascus gabriellae*; and the common marmoset, *Callithrix jacchus*). Using the assay that has been previously established for Schlafen11 [15], we transfected into 293T cells each of these Schlafen11-expressing plasmids, along with a plasmid encoding an HIV-1 proviral clone (pNL4-3.Luc.R^+^E^-^). 293T cells are naturally hypomorphic for Schlafen11 expression compared to other human cell types ([15] and S1 Fig). After 48 hours, cellular extracts were evaluated by immunoblotting for the production of Schlafen11 and an HIV-1 protein (p24). As shown previously, human Schlafen11 was able to reduce production of p24 encoded by the HIV-1 genome (Fig 2A). Bonobo Schlafen11 had similar activity, consistent with the close evolutionary relationship of human and bonobo. However, both gibbon and marmoset Schlafen11 had dramatically increased activity in this assay, with marmoset Schlafen11 consistently blocking almost all HIV-1 p24 (Fig 2A). None of the Schlafen11 proteins had any effect on the protein levels of a cellular control, GAPDH. Thus, the amino acid differences that have accumulated in the Schlafen11 proteins encoded by different species affect their ability to inhibit HIV-1 protein production.

### Schlafen11 affects viral protein production outside of the context of infection

We tested whether Schlafen11 was active against a single HIV-1 protein product, Gag-Pol, independent of other viral components. We transfected into 293T cells a plasmid encoding each primate Schlafen11 along with two plasmids encoding HIV-1 Gag-Pol and Rev (needed for nuclear export of the Gag-Pol transcript). After 48 hours, cell extracts were probed for Schlafen11 and HIV-1 p24 protein production. The species-specific differences in Schlafen11 were the same as before, where gibbon and marmoset Schlafen11 had increased activity at inhibiting HIV-1 p24 production compared to human and bonobo Schlafen11 (Fig 2B). We confirmed that the production of unprocessed Gag was also affected (S2 Fig). Therefore, if the species-specific differences in Schlafen11 activity reflect differential susceptibility to a viral antagonist, this antagonist must be encoded within the Gag-Pol polyprotein or Rev. We next tested the ability of different primate Schlafen11 proteins to inhibit Gag-Pol production from other retroviral genomes: murine leukemia virus (MLV) and feline immunodeficiency virus (FIV). Plasmids expressing MLV and FIV Gag-Pol were co-transfected with plasmids expressing various primate Schlafen11. Immunoblots were used to monitor the production of p30 by MLV and p26 by FIV. Once again, the gibbon and marmoset Schlafen11 were more potent at diminishing protein production than human and bonobo Schlafen11 (Fig 2C and 2D). In these transfection-based experiments, Schlafen11 homologs were sometimes expressed at slightly different levels, although increased expression was not noted to track with the increased suppression of viral protein production. To attempt to quantify the relative activity of the different Schlafen11 homologs, we normalized the amount of viral protein produced to the relative amount of Schlafen11 (Fig 2E, increasing Schlafen11 activity from blue (least active) to red (most active), see materials and methods for an explanation of normalization process). In general, the quantification procedure confirms that marmoset and gibbon Schlafen11 are the most active homologs. We also find that when HIV-1, MLV, and FIV are packaged in cells that are expressing Schlafen11, fewer infectious virions result (Fig 2F). In both the immunoblotting and packaging assays, we see a nearly identical species-specific pattern of inhibition for all viruses, with marmoset and gibbon Schlafen11 being more potent inhibitors than human Schlafen11. Thus, the ability of Schlafen11 to suppress viral protein production is specific to each host species but, curiously, virus independent.

### Schlafen11 blocks protein production from non-viral transcripts

**Fig 3:**
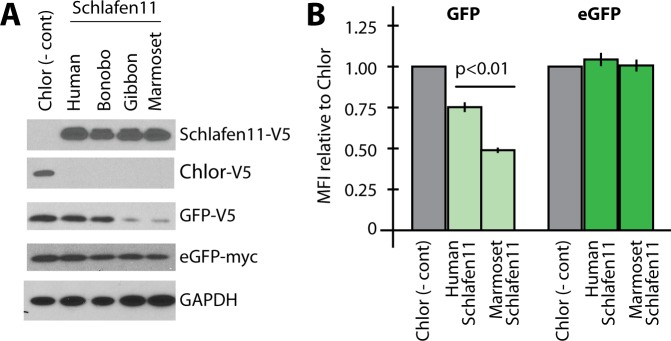
Schlafen11 reduces protein production from non-viral transcripts. **(A)** Schlafen11 or Chlor (negative control) expressing plasmids were cotransfected into 293T cells along with plasmids expressing either a V5-tagged GFP or myc-tagged eGFP. Total protein was harvested 48 hours post transfection and probed for the indicated proteins. The blot is representative of 3 independent experiments. **(B)** Either GFP or eGFP (untagged) expressing plasmids were cotransfected along with plasmids encoding Schlafen11 as in (A). 48 hours post transfection, flow cytometric analysis was performed to measure the mean fluorescence intensity (MFI) of the GFP signal. All values reported are relative the the Chlor control. Error bars are standard error from 3 independent experiments.

Given that Schlafen11 restricted viral protein production outside of the context of infection, we wondered if viral proteins were necessary at all. To explore this, we tested the activity of primate Schlafen11 proteins against non-viral proteins. Schlafen11 was previously found to affect the translation of GFP but not the codon optimized version of this gene (eGFP), but these experiments were performed in the context of co-transfection with an HIV-1 proviral clone [15]. We transfected mammalian expression plasmids encoding GFP and eGFP into 293T cells only along with plasmids encoding primate Schlafen11 proteins. After 48 hours, cellular extracts were probed for GFP/eGFP protein production by western blot. We were able to differentiate these two forms of GFP on a western blot by adding a V5 tag to GFP and a codon-optimized myc tag to eGFP. The identical species-specific pattern of Schlafen11 inhibition seen for HIV-1 proteins was also observed for GFP, with marmoset and gibbon Schlafen11 having the most extreme inhibitory effect (Fig 3A). As expected, there were no significant differences in eGFP protein production in the presence of any version of Schlafen11. We confirmed these results in a flow cytometry based assay, where GFP or eGFP (both without a tag) were coexpressed with either Schlafen11 from human or marmoset, or with chloramphenichol acetyltransferase (Chlor) as a negative control. Cells showed less fluorescence in the presence of marmoset Schlafen11 than human Schlafen11, but only for GFP and not for eGFP (Fig 3B). Overall, since there are no virus components of any type in these experiments, this strongly suggests that the species-specific differences in activities of Schlafen11 have little to do with a specific viral target, or with sensitivity to a viral antagonist. It appears that some primate versions of Schlafen11 are simply fundamentally more active than others in their ability to inhibit protein production from non-codon optimized transcripts. It is difficult to speculate further, since the mechanism of Schlafen11 is not yet well understood.

### Schlafen11 inhibits the accumulation of human proteins

**Fig 4:**
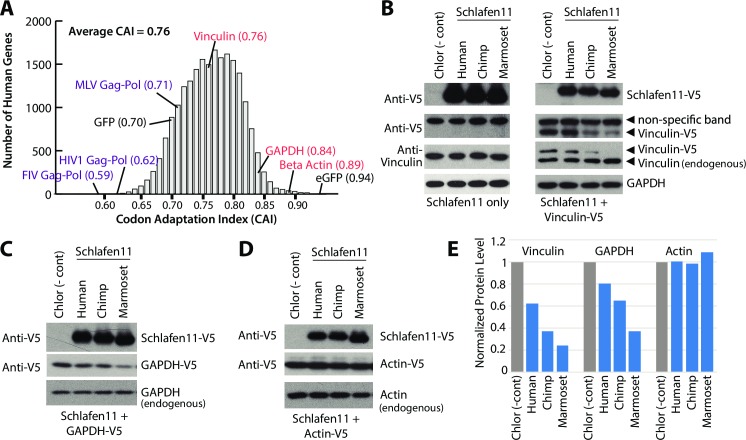
Schlafen11 inhibits production of human proteins. **(A)** Codon adaptation index (CAI) is plotted for all human genes, and for select other genes (viral and GFP/eGFP) used in this study. Human genes analyzed in this study are indicated in red, viral genes in purple. **(B,C,D)** Plasmids encoding the indicated Schlafen11-V5 proteins, or Chlor, were cotransfected into 293T cells (B) with or without a Vinculin-V5 expressing plasmid, (C) with a GAPDH-V5 expressing plasmid, or (D) with an Actin-V5 expressing plasmid. Cell lysates were subject to immunoblotting as indicated. Blots are representative from two independent experiments. **(E)** Quantification of the V5-tagged proteins detected in panels B-D. All bands are normalized to the quantification of endogenous GAPDH.

Given that Schlafen11 can reduce protein production from gene transcripts outside of the context of infection, it stood to reason that human genes could also be susceptible to Schlafen11. To test this, we wished to choose a human gene that was relatively non-optimal in its usage of codons. We first calculated the codon adaptation index (CAI) of all human genes (Fig 4A). We then overlaid onto this distribution the CAI of each gene analyzed in this study (human or otherwise). GAPDH, used as a control in this study, and eGFP are in the upper tail of the distribution (most codon optimized). We have found that both of these genes are resistant to the effects of Schlafen11. Notably, eGFP has a CAI that is even higher than any actual human gene. On the other hand, the viral genes and GFP, all of which we have found are susceptible to Schlafen11, fall in the lower half of the distribution (least codon optimized).

To test whether Schlafen11 can inhibit the production of human proteins, we chose three human proteins for which good antibodies exist: Actin, GAPDH, and Vinculin. Actin and GAPDH have a very high CAI (Fig 4A), which we hypothesized would make them more resistant to Schlafen11. Conversely, Vinculin has a lower CAI (Fig 4A), which should cause it to be more susceptible to Schlafen11. We first tested Vinculin by transfecting plasmids encoding Schlafen11 from several primate species (in this case, human, chimpanzee (*Pan troglodytes*), and marmoset) alone or with a plasmid encoding V5-tagged Vinculin. Cell extracts were harvested 48 hours later and subject to immunoblotting with a V5 antibody. We found that human Schlafen11 only slightly affected protein levels of Vinculin-V5 (Fig 4B and 4E). However, chimpanzee and marmoset Schlafen11 significantly decreased levels of Vinculin-V5 (Fig 4B and 4E). No Schlafen11 proteins affected the levels of endogenous Vinculin, as determined by immunoblotting with an anti-Vinculin antibody (Fig 4B). This can perhaps be explained by the fact that this protein pre-existed in the cell, while the V5-tagged version did not, and by the half-life of Vinculin which is 12 hours [39]. We cannot rule out that overexpression of genes from plasmids or viral genomes makes them generally more susceptible to Schlafen11, because we have yet to detect an effect on an endogenous human protein. However, this experiment does tell us that there is nothing particularly special about viral genes over host genes in this system.

We next tested a plasmid-expressed, V5-tagged version of GAPDH, which has a much higher codon adaptation index (Fig 4A). The accumulation of GAPDH-V5 was somewhat affected by Schlafen11 (particularly by the marmoset Schlafen11) (Fig 4C and 4E). Again, the endogenous version of GAPDH was quite stable (Fig 4B and 4C). Finally, Actin-V5, which has the highest CAI of the three host genes tested, was not affected by any Schlafen11 (Fig 4D and 4E). We conclude that Schlafen11, particularly the version encoded by some non-human primates, generally blocks protein accumulation, including the accumulation of human proteins. However, genes with higher codon adaptation indices (more optimal codon usage) appear to be more protected from these effects. We cannot rule out that other genetic features besides codon usage may also be contributing to susceptibility to Schlafen11.

### Schlafen11 is expressed in most tissues and is active at physiologically relevant levels

**Fig 5:**
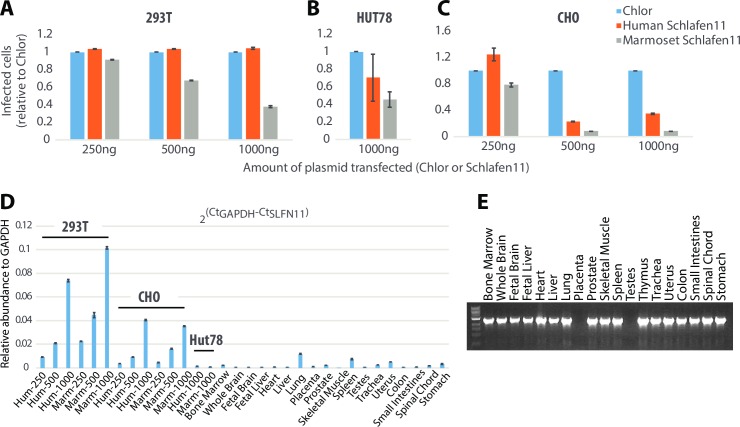
Schlafen11 is expressed in various human tissues and is active at physiologically-relevant levels. **(A)** 293T cells were cotransfected with viral packaging plasmids required to create VSV G-pseudotyped MLV along with increasing amounts of pcDNA6.2 encoding the indicated Schlafen11 or Chlor. Pseudotyped MLV produced was measured on 293T cells and the relative amount of virus production (standardized to Chlor) was determined. **(B)** Identical to (A), except performed in HUT78 cells (a T-cell line). Only the results from 1000ng is shown as other experiments did not yield replicable results. **(C)** Identical to (A), except performed in Chinese Hamster Ovary (CHO) cells. **(D)** Quantitative PCR was used to measure *SLFN11* expression levels in cDNA from transfection conditions in panels A-C, and from a set of human tissues. Error bars represent the standard error of three independent replicate experiments. **(E)** *SLFN11* was amplified by non-quantitative PCR from a cDNA panel representing the human tissues indicated.

To ensure that the above observations were not specific to 293T cells, we performed Schlafen11 assays in Hut78 (human T cells; representing the primary replication sites for HIV-1) and CHO (Chinese hamster ovary) cells. Into each cell line, we cotransfected MLV viral packaging plasmids along with increasing amounts of the mammalian expression vector encoding human or marmoset Schlafen11 (or Chlor as a negative control). The relative amount of MLV particles produced (standardized to Chlor) was determined by titration on 293T cells. We found that marmoset Schlafen11 was able to decrease viral production in 293T cells even at the lowest amount of transfection (250ng, Fig 5A). We found a similar species-specific difference in the ability of human and marmoset Schlafen11 to reduce viral production in Hut78 (Fig 5B) and CHO cells (Fig 5C) as well. Direct comparison of the levels of inhibition between cell lines cannot be made, because some of these cell lines are more readily transfectable than others. This can be seen in results from a qRT-PCR experiment, where Schlafen11 expressed in 293T and CHO cells with 250ng of plasmid is greater than the amount of Schlafen11 expressed in Hut78 cells with 1000ng of plasmid (Fig 5D). Keeping this in mind, it does appear that Schlafen11 is particularly active in CHO cells and interestingly, even human Schlafen11 showed an ability to reduce viral production in CHO cells (Fig 5C), compared to no effect in 293T cells (Fig 5A), even though expression was not higher in CHO cells (Fig 5D). In all three cell lines, marmoset Schlafen11 continued to be a more potent than human Schlafen11. Therefore, we have shown that Schlafen11 functions in multiple cell lines, including T cells, and that the species-specific pattern of Schlafen11 activity is constant regardless of the cellular context in which it is functioning.

Next, we wanted to assess physiological relevance of the Schlafen11 expression levels achieved in our experiments. In order to assess Schlafen11 expression patterns *in vivo*, we first performed non-quantitative PCR on cDNA libraries constructed from a number of human tissues (Fig 5E). We found that Schlafen11 appears to be expressed in all tissues tested except for placenta and testes. We then performed quantitative PCR on cDNA from cells transfected with Schlafen11 (the experimental conditions from Fig 5A–5C), and the human cDNA libraries. We found, as expected, that transfection induced much higher levels of mRNA expression than is found naturally in human tissue. However, at the lowest transfection levels in 293T, CHO, and HUT78 cells, we saw a level of Schlafen11 expression that was comparable to that found in natural tissues. Therefore, we conclude that Schlafen11 is active at physiologically relevant levels. It should be noted that the tissue panel analyzed in panels D and E is made from tissues not known to be interferon stimulated, so these data may represent the low end of the physiological expression level for this gene.

### Positive selection of the *SLFN11* gene does not tailor relative activity of primate Schlafen11 proteins

**Fig 6:**
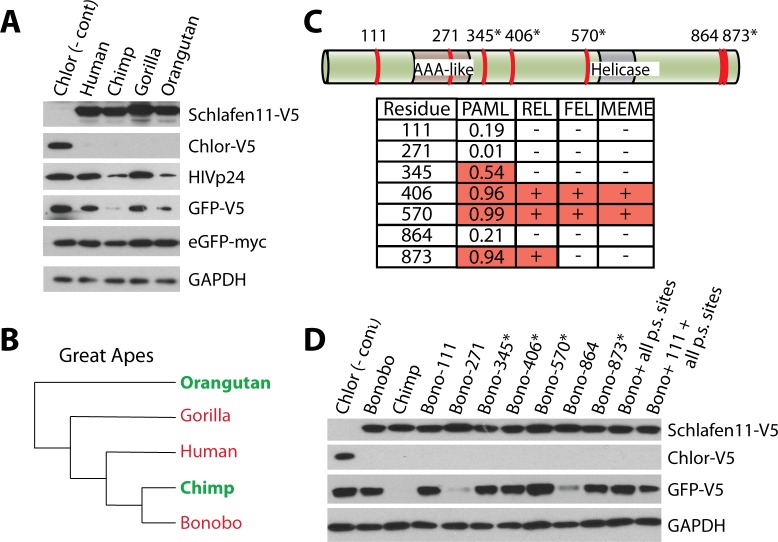
Sites under positive selection do not govern species-specific differences in activity of Schlafen11. **(A)** Plasmids encoding HIV-Gag-Pol, HIV-Rev, GFP, and eGFP were cotransfected into 293T cells along with a plasmid encoding the indicated great ape Schlafen11, or Chlor as a negative control. Cell lysates were subject to immunoblotting. **(B)** A phylogeny of the great apes is shown, with the activity of the Schlafen11 of each species indicated. Those in green have strong ability to suppress protein production when expressed in human cells, while those in red are weaker. **(C)** The seven amino acid positions that differ between chimpanzee and bonobo Schlafen11 are illustrated on a protein schematic (see S3 Fig for an alignment). Asterisks indicate sites under positive selection. The table shows the posterior probability of each site being under positive selection (PAML), as well as which sites were determined to be under positive selection by the other three tests. We liberally included position 345 in this group because it was detected in our PAML analysis with a posterior probability of >0.5, providing only weak support for selection (i.e. there is a 54% chance that this codon was correctly assigned to the dN/dS > 1 site class by PAML). **(D)** Site directed mutagenesis was performed on bonobo Schlafen11 at the indicated sites, changing them to the amino acids encoded by chimpanzee Schlafen11. Plasmids encoding wild-type or mutant Schlafen11, or chloramphenicol acetyltransferase (Chlor) as a negative control, were cotransfected into 293T cells along with a plasmid encoding GFP. Cell lysates were subject to immunoblotting. “p.s. sites” refers to positively selected sites and, in these two clones, all positions under positive selection were simultaneously changed in bonobo Schafen11 to the residues encoded in chimpanzee Schlafen11. The blots in panels A and D are representative of two independent experiments.

Many of the active primate Schlafen11 proteins tested have a large number of amino acid differences from the non-active Schlafen11 proteins (e.g. 123 non-synonymous substitutions between human and marmoset Schlafen11). To further assess the evolutionary divide between active and non-active Schlafen11 proteins, we took a focused look at the great apes. We cloned Schlafen11 from human, chimpanzee, gorilla (*Gorilla gorilla*) and orangutan (*Pongo abelii*) into expression plasmids. We tested these great ape Schlafen11s by cotransfecting them along with plasmids encoding HIV-1 Gag-Pol, GFP, and eGFP. This allowed us to look at the effects of each of the Schlafen11 proteins on three different target proteins in a single experiment (HIV-1 p24, GFP, and eGFP). Despite the close relationship of these species, and the fact that their Schlafen11 proteins are all greater than 94% identical, there were stark species-specific differences in the inhibition of protein production. Notably chimpanzee and orangutan Schlafen11 were more active than human or gorilla Schlafen11 in this assay (Fig 6A), and inhibited both p24 and GFP protein accumulation. Neither affected eGFP or GAPDH, the more codon optimized targets. Together with bonobo Schlafen11 tested previously, we can now superimpose the activity of different Schlafen11 proteins onto a cladogram of the great apes (Fig 6B). Interestingly, there is no clustering of active (green) and inactive (red) Schlafen11 proteins, and therefore this protein has been quite dynamic even in this recently diverged clade of primates.

Because we have performed an analysis of the codons targeted by positive selection in the *SLFN11* gene, we had the opportunity to ask whether amino acid differences at sites under selection dictate the increased activity observed for some primate Schlafen11 proteins. We chose to do site-directed mutagenesis between chimpanzee and bonobo Schlafen11 because of their close phylogenetic relationship (99% identity) as well as differential activity, where chimpanzee Schlafen11 is active (Figs 4 and 6A) and bonobo Schlafen11 is not (Figs 2 and 3A). There are only 7 nonsynonymous differences between the bonobo and chimpanzee *SLFN11* genes, and 4 of these differences are in codons detected to be evolving under positive selection (Fig 6C, sites under positive selection are denoted by asterisks and support for positive selection is given in table below). We mutated each of these seven sites in bonobo Schlafen11 to encode the amino acid found in chimpanzee Schlafen11. We did not observe any increase in activity of bonobo Schlafen11 (resulting in reduced GFP-V5 protein production in a co-transfection assay) when any of the sites under positive selection were mutated, either individually or all together (Fig 6D). Instead, it was when two of the remaining three differences between chimpanzee and bonobo Schlafen11, sites 271 or 864, were muted that we saw a dramatic increase in activity by bonobo Schlafen11 (Fig 6D). These sites are located in the AAA-like domain and at the extreme C-terminus of Schlafen11 (Fig 6C). Interestingly, we noticed that one of these sites, 271, is polymorphic in bonobos, suggesting this species may maintain *SLFN11* alleles with differential activity. No single point mutant made in a human Schlafen11 background resulted in an increase in activity (S3 Fig). While the Schlafen11 proteins encoded by different primate species have differential potencies at suppressing protein production, this pattern does not seem to be resulting from recurrent positive selection of particular codons in this gene.

## Discussion

*SLFN11* is an interferon-stimulated gene (S1 Fig) [15,38,40] that encodes a protein with suppressive effects on protein accumulation, suppressing levels of both host and viral proteins. It is curious to consider how HIV-1 bypasses the effects of Schlafen11. However, because the human Schlafen11 is relatively weak in all of the contexts that we have analyzed, HIV-1 may not require an antagonist. In general, Schlafen11 proteins block protein production from genes containing non-optimized codons to a greater extent than from genes with more optimized codons, although further investigation may reveal other determinants of Schlafen11 suppression. While human Schlafen11 does have mild effects on protein production, these effects are dramatically pronounced in some nonhuman primate versions of Schlafen11. For instance, Schlafen11 from gibbon, marmoset, chimpanzee, and orangutan are all much stronger inhibitors. Schlafen11 from human, bonobo, and gorilla are much weaker. It remains unknown why there are consistent species-specific differences in the potencies of primate Schlafen11. For instance, marmoset Schlafen11 is a potent suppressor of protein production for all targets tested: viral proteins (HIV, FIV, MLV), non-viral proteins (GFP), and even host proteins (Vinculin and GAPDH). On the other hand, human Schlafen11 is a weak suppressor of all of these proteins. We can only speculate that perhaps there is a cellular cost to Schlafen11, and that in certain species the genes encoding these proteins have been selected in favor of inactivating mutations that reduce these costs when pathogen-related pressure is not high. Of course, it may also be relevant that primate genomes contain an entire family of *SLFN* gene paralogs located in a single genomic cluster [41], although the functions of others have not been explored. It is possible that functional decay is happening in *SLFN11* in some species because the antiviral role has been refined in another gene paralog in that species.

The gene encoding Schlafen11 has evolved under recurrent positive selection, but this doesn’t appear to have tailored the relative potencies of different primate versions. Instead, this positive selection may be a result of selection imposed by one or more unidentified viral antagonists. Viruses from the genus *Orthopoxvirus* encode a truncated *SLFN*-like gene in their genomes, the result of a horizontal gene transfer event from a mammalian host [41]. This viral protein could act as a mimic to restrict the action of host Schlafen proteins, possibly by inhibiting Schlafen multimerization or via another mechanism. Mimicry of host proteins is a common strategy for immunological evasion employed by pox viruses [42–44]. Given that orthopoxviruses infect a number of primate species [45], they could theoretically have driven an arms race dynamic with *SLFN* genes including *SLFN11*. It is also possible that the signature of positive selection in *SLFN11* is unrelated to viral antagonism. Indeed, some mammalian *SLFN* genes have been implicated in sperm-egg incompatibility. For example, crossing mice with mismatched *SLFN* loci results in death of the embryo soon after implantation, referred to as DDK syndrome [46]. Similarly, the *SLFN* gene locus has also been implicated in the unequal segregation of chromatids during the second meiotic division [47]. Both sperm-egg interactions and meiotic drive can result in a strong signature of recurrent positive selection due to arms race dynamics [48–50]. Finally, it is also possible that the signatures of positive selection are due to selective pressures from both of these forces (sexual and pathogen conflict) simultaneously.

## Materials and Methods

### Primate sequences and samples

Primate *SLFN11* gene sequences were retrieved from available primate genome projects, using the BLAT function on the UCSC genome browser [51]. The remaining *SLFN11* gene sequences were obtained by direct sequencing of cDNA libraries produced from primary or immortalized primate fibroblast cell lines. Briefly, cells were grown in DMEM (Cellgro) supplemented with 15% FBS (Gibco) at 37°C and 5% CO_2_. RNA was extracted using the AllPrep DNA/RNA extraction kit (QIAGEN). cDNA libraries were generated using SuperScript III first strand synthesis kit (Invitrogen). PCR was performed using PCR SuperMix High Fidelity (Invitrogen). PCR products were directly sequenced. Each primate sequence was used as a query to search the human genome, and human *SLFN11* gene was returned as the top hit. *SLFN11* gene sequences have been deposited in GenBank (accession numbers KY204401-KY204411). Sequences aligned to using the MUSCLE algorithm were used for downstream evolutionary analysis [52]. The human Schlafen11 sequence used to query UCSC was obtained from NCBI (accession number NM_001104587). The RNA used to sequence the other primate sequences was obtained from the following cell lines: Bonobo (*Pan paniscus*, Coriell PR00748), Bornean Orangutan (*Pongo pygmaeus*, Coriell PR00650), Pileated Gibbon (*Hylobates pileatus*, Coriell PR00243), Red-Cheeked Gibbon (*Nomascus gabriellae*, Coriell PR00381), Crab-Eating Macaque (*Macaca fasicularis*, 103–06, gift from Welkin Johnson), Black Mangabey (*Lophocebus albigena*, Coriell PR01215), Olive Baboon (*Papio anubis*, Coriell PR00978), Talapoin (*Miopithecus talapoin*, Coriell PR00716), Wolf’s Guenon (*Cercopithecus wolfi*, Coriell PR01241), Colobus (*Colobus guereza*, Coriell PR00980), Squirrel Monkey (*Saimiri sciureus*, Coriell PR00603). Additional samples used to amplify functional clones are common marmoset (*Callithrix jacchus*, Coriell GM07404), chimpanzee (*Pan troglodytes*, Coriell NS06006), gorilla (*Gorilla gorilla*, Coriell PR00280) and orangutan (*Pongo abelii*, Coriell PR01052). The human clone was from [15].

### Detection of positive selection

Detection of recurrent positive selection was carried out using several commonly used programs. The codeml program in the PAML 4.3 software package was used to fit the *SLFN11* multiple sequence alignment to two codon models: M8 and M8a [53]. M8a is a neutral model where all codons are constrained to evolve with a dN/dS ≤ 1. M8 is a model of positive selection (M8) where some codons are allowed to evolve with a dN/dS > 1. Using a likelihood ratio test, we found that the positive selection model was a significantly better fit to the data (p<0.0001). In model M8, 36 codons were assigned to the dN/dS > 1 class with high posterior probability (p>0.9) by Bayes Empirical Bayes (BEB) analysis (S1 Table). Two ω_0_ seed values (0.4 and 1.5) gave similar results, as did the f61 and f3x4 codon frequency models. The *SLFN11* alignment was also analyzed for positive selection using REL, FEL, and MEME as implemented in Datamonkey [54–57]. Sites with dN/dS > 1 identified by each of these methods are indicated in S1 Table. The cutoff was p<0.01 for FEL, Bayes factor > 50 for REL, and p<0.09 for MEME in S1 Table.

### Plasmids and viruses

Genes encoding primate Schlafen11, GFP, eGFP, Vinculin, Actin, and GAPDH were TA-cloned into pcDNA6.2/GW/D-TOPO mammalian expression vector (Invitrogen). eGFP was cloned with a codon-optimized myc tag on the C-terminus followed by stop-codon to prevent translation of the V5 tag encoded in pcDNA6.2. All other genes were V5 tagged, as this was a property of the vector used (see product literature for pcDNA6.2). Chlor-V5 was provided by Invitrogen in the TA-cloning kit as a control vector. pNL4-3.Luc.R^+^E^-^ was obtained from the National institutes of health AIDS reference and reagents program. Plasmids encoding HIV-1 Gag-Pol (pMDLg/pRRE; from Addgene), HIV-1 REV (pRSV-REV; from Addgene), NB-MLV Gag-Pol (CS2-mGP) [58], and FIV Gag-Pol (pFP93)[59].

### Transfections and immunoblotting

293T cells (ATCC CRL-3216) were maintained in Dulbecco’s modified eagles media (DMEM) supplemented with L-glutamine, pen/strep, and 10% FBS. Cells were seeded into 6-well dishes at a concentration of 800,000 cells/well. About 24 hours after seeding, cells were transfected with 2ug of a plasmid encoding the indicated Schlafen11 or chloramphenichol acetyltransferase (Chlor) as a negative control, along with plasmids encoding the pNL4-3.Luc.R^+^E^-^ (1000ng), HIV-1 Gag-Pol (500ng) + RSV-Rev (250ng), NB-MLV Gag-Pol (1000ng), GFP (500ng), human Vinculin (500ng), human GAPDH (500ng), human Actin (500ng), and/or eGFP (50ng). 48 hours post transfection, cells were lysed in the dish using RIPA buffer. Lysate was loaded onto a 10% SDS-PAGE gel. Protein was transferred to a polyvinyl membrane and blocked with a TBS solution containing 5% milk and 0.1% TWEEN20. Immunoblotting was performed using the following primary antibodies rabbit-anti-V5 antibody (SantaCruz Biotech G-14, sc83849), rabbit-anti-GAPDH (Cell Signaling, 14C10), rabbit-anti-myc (Abcam, ab9106), mouse-anti-HIV-1 p24 (NIH Aids Reagents, 183-H12-5C), and rabbit-anti-MLV p30 (Abcam, ab100970). HRP conjugated secondary antibodies used: goat-anti-mouse (Thermo, 32430), goat-anti-rabbit (Thermo, 32460), donkey-anti-goat (SantaCruz biotech, sc-2020). Protein bands were visualized using ECL-prime (GE, RPN2236). Quantification of westerns was performed as follows. Western blot images were loaded into imageJ. The bands corresponding to GAPDH, Schlafen11, and the viral protein (p24, p30, or p26) were quantified using standard techniques. The relative amount of viral protein was normalized using the following equation:

p′=(QGagPol−SLFNQGagPol−CAT)(QSLFN11QGAPDH)p

Where *Q*_*GagPol–SLFN*_ is the quantification of the viral band in the Schlafen11 lane, *Q*_*GagPol–CAT*_ is the quantification of the viral band in the Chlor lane, *Q*_*SLFN*11_ is the quantification of the Schlafen11 band, and *Q*_*GAPDH*_ is the quantification of the GAPDH band, and *p* is the numerator calculated for the human Schlafen11 experiment.

### Virus packaging assays

Pseudotyped virus was produced using a recombinant retroviral packaging system, which utilized a VSV-G glycoprotein, a retroviral Gag-Pol, and a GFP flanked by UTRs specific for each retrovirus. The amount of each component, and the plasmid name follows. See plasmids and viruses section for more details on plasmids. Pseudotyped MLV was produced by cotransfecting 0.2ug pC-VSVG (VSVG encoding plasmid), 1ug CS2-mGP (NB-MLV Gag-Pol encoding plasmid), and 2ug pLXCG (eGFP). Pseudotyped HIV was produced by cotransfecting 0.3ug pMD2.G (VSVG; addgene plasmid #12259), 0.5ug pMDLg/pRRE (HIV Gag-Pol; addgene plasmid #12259), 0.25ug RSV-Rev (HIV Rev; addgene plasmid #12253), and 1ug pRRLSIN-GFP (eGFP; addgene plasmid #12252). Pseudotyped FIV was produced by cotransfecting 0.2ug pMD2.G, pGinSin (GFP), and pFP93 (FIV Gag-Pol derived from the 34TF10 molecular clone). FIV reagents were a gift from Dr. Eric Poeschla. Either 1/10th, 1/2, or the full amounts of plasmid reported above for each viral system was cotransfected into 293T cells along with 2ug Schlafen11 or Chlor plasmid. 48 hours post transfection, supernatant was collected and titrated on 293T cells. 2.5x10^5 293T cells were plated in a 24 well dish. 24 hours after plating, these cells were infected with 25ul of virus-containing supernatant. The media was treated with 10ug/mL polybrene and centrifuged for 1 hour at 1,200 rpm. 24 hours post infection, cells were measured for GFP expression by flow cytometry.

### Site directed mutagenesis

Site directed mutagenesis of plasmids was performed using PFU turbo (Agilent technologies) and primers with a built in mutation. Primer sequences available upon request. 18 cycles of PCR was performed with the following conditions: 1min at 95°C, 1min at 57°C, 9min at 65°C. The reaction was digested with DPN-I (NEB) for 4hrs followed by transformation into competent DH5α cells (Invitrogen). Correct mutagenesis was confirmed by Sanger sequencing.

### Flow cytometry analysis

48 hours after transfection, cells were trypsinized and resuspended in PBS containing 1% paraformaldehyde for 10 minutes. Cells were washed 3x with PBS and then resuspended in PBS + 2% FBS + 1mM EDTA. Flow cytometry was performed on a BD LSRFortessa Flow Cytometer. Analysis of mean fluorescence intensity (MFI) or percent GFP-positive was performed using FlowJo version 10.1.

### CAI calculations

To calculate CAIs, the coding sequences of all human genes (GRCh38) were downloaded from Ensembl. These sequences were then filtered to the 21,789 longest isoforms of genes with well-formed ORF sequences and Ensembl biotype annotation indicating the production of actual protein products. CAIs for all human genes, and for our genes of interest, were then calculated using the original formula for CAI given by Sharp and Li [60]:

$$CAI(g)={\left(\prod_{k=1}^{L}w_{k}\right)}^{1/L}$$

where L is the length of the sequence, and w_k_ is the weight of the k-th codon calculated by:

$$w_{i,j}=\frac{x_{i,j}}{y_{j}}$$

Where x_i,j_ is the count of the *i*-th codon of the *j*-th amino acid in some reference set and *y*_*j*_ is the count of the most used codon of amino acid *j* in the reference set. We chose the reference set by two different methods. First, we used the recommendation by Sharp and Li, and chose the top 1% of genes found to be most highly expressed. To determine this set of genes, we used publicly available, two replicate HEK293T mRNA-Seq data posted to the Gene Expression Omnibus (GSM1440304, GSM1440305). Data was cleaned, mapped to the longest isoforms with Bowtie2, and used to estimate relative gene expression with RSEM. We also naively chose the entire set of accepted longest isoforms as the reference set. No notable difference in the distribution of CAIs was obvious between these two methods. The results reported use the naïve longest-isoform reference set.

### Quantitative PCR

Total RNA was harvested from cells transfected with indicated amounts of Schlafen11-V5-pcDNA6.2 using phenol-chloroform-isoamyl alcohol (Sigma). RNA was treated with Turbo DNaseI (Thermo-Fisher) and re-extracted with phenol-chloroform-isoamyl alcohol. RNA was reverse transcribed using SuperScript III First-Strand Synthesis System (Thermo-Fisher), using 250ng RNA per reaction and random hexamer primers. The tissue panel shown in Fig 5 was obtained from Clontech. Schlafen11 was quantified using the following primers: S11-F 5’CCTCCCCTTAGCAGACCAGT3’, S11-R 5’TTCCCCGAAAGAAAGGTTG3’, GAPDH-F 5’ACCGTCAAGGCTGAGAACGG3’, GAPDH-R 5’GTGGTGAAGACGCCAGTGGA3’. When quantifying in CHO cells, the GAPDH primers used were 5’GGCTGCCCAGAACATCATCC3’ 5’CTTCCCGTTCAGCTCTGGGA3’. Delta-Ct values were calculated by subtracting the Ct value of GAPDH from the Ct value of SLFN11. Variance of this measurement was calculated using propagation of error.

## Supporting Information

S1 Table Codons in SLFN11 identified with dN/dS > 1 in four different tests for positive selection. (TIF)

S1 Fig The expression of Schlafen11 is stimulated by interferon β in 293T cells. Four common human cell lines were treated with 1x10^6 IU/mL of interferon β-1b for 24 hours before cell lysates were harvested. RNA was purified from these extracts and reverse transcribed. A fragment of the *SLFN11* transcript was then amplified by PCR. It can be noted that in the absence of treatment, 293T cells are hypomorphic for Schlafen11 compared to all other cells tested, as noted in [15].
(TIF)

S2 Fig Unprocessed HIV-1 Gag is affected by Schlafen11. Image of a western blot showing that unprocessed Gag is affected by marmoset Schlafen11 in our experiments.
(TIF)

S3 Fig **No single mutation in human Schlafen11 conveys the ability to inhibit translation** (A) A multiple sequence alignment of human, bonobo, and chimpanzee Schlafen11 is shown. Differences are highlighted as indicated. (B) Site directed mutagenesis was used to change indicated residues in human Schlafen11 to those found in chimpanzee Schlafen11. Plasmids encoding these modified proteins (or chimpanzee Schlafen11 as a positive control; chloramphenichol acetyltransferase (Chlor) as a negative control) were then co-transfected into 293T cells along with a plasmid encoding GFP. Cell extracts were subject to immunoblotting and GFP protein levels were used as a read-out of translational suppression. All numbers across the top refer to positions in the human protein which were mutated to match the residue found in the chimpanzee protein.
(TIF)
