# Supplementary figures and images for: Non-human Primate Schlafen11 Inhibits Production of Both Host and Viral Proteins
Source: PLoS Pathog. 2016 Dec 27;12(12):e1006066. doi: 10.1371/journal.ppat.1006066 (PMC5189954; doi:10.1371/journal.ppat.1006066)

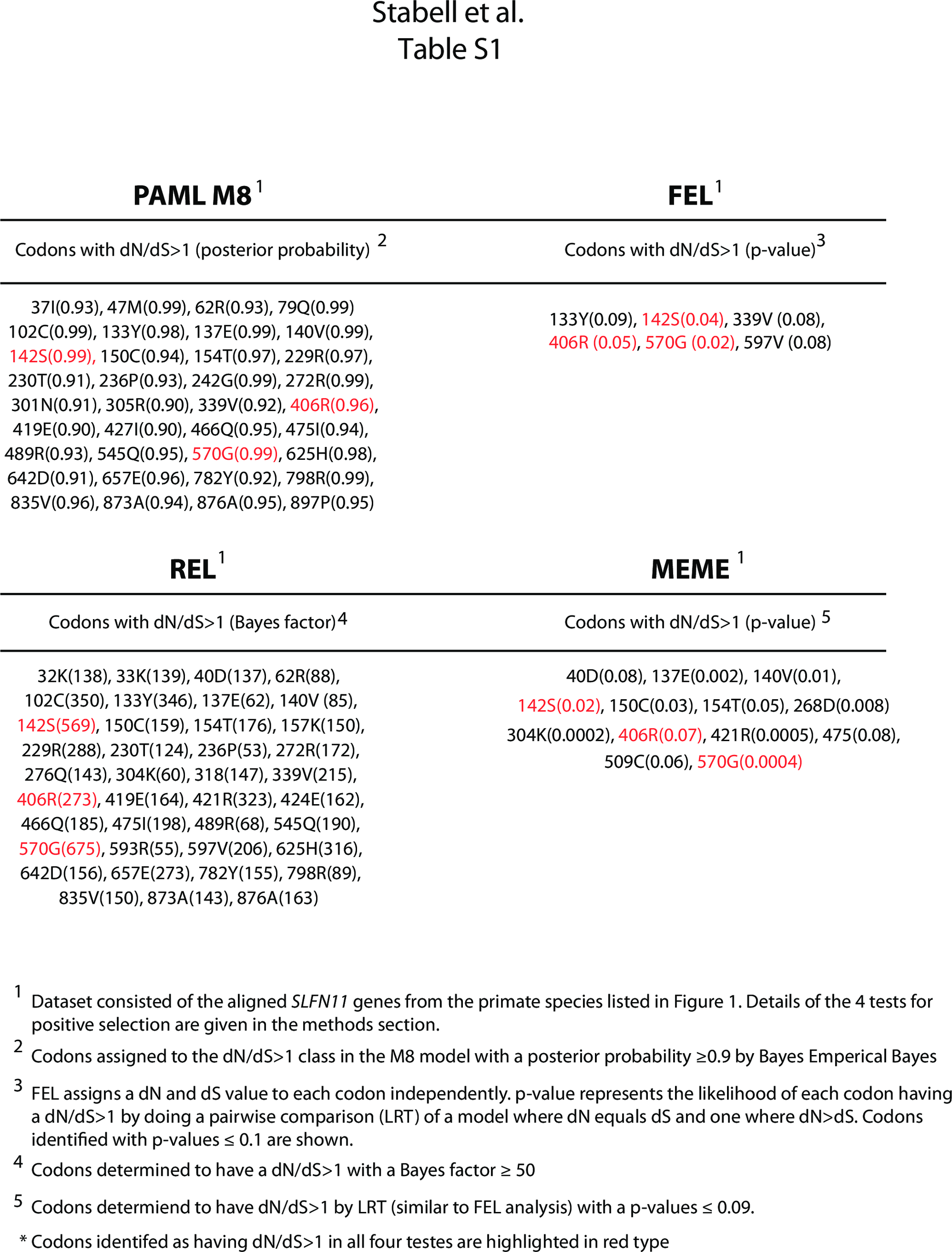

Supplement: S1 Table — (TIF) [file ppat.1006066.s001.tif]

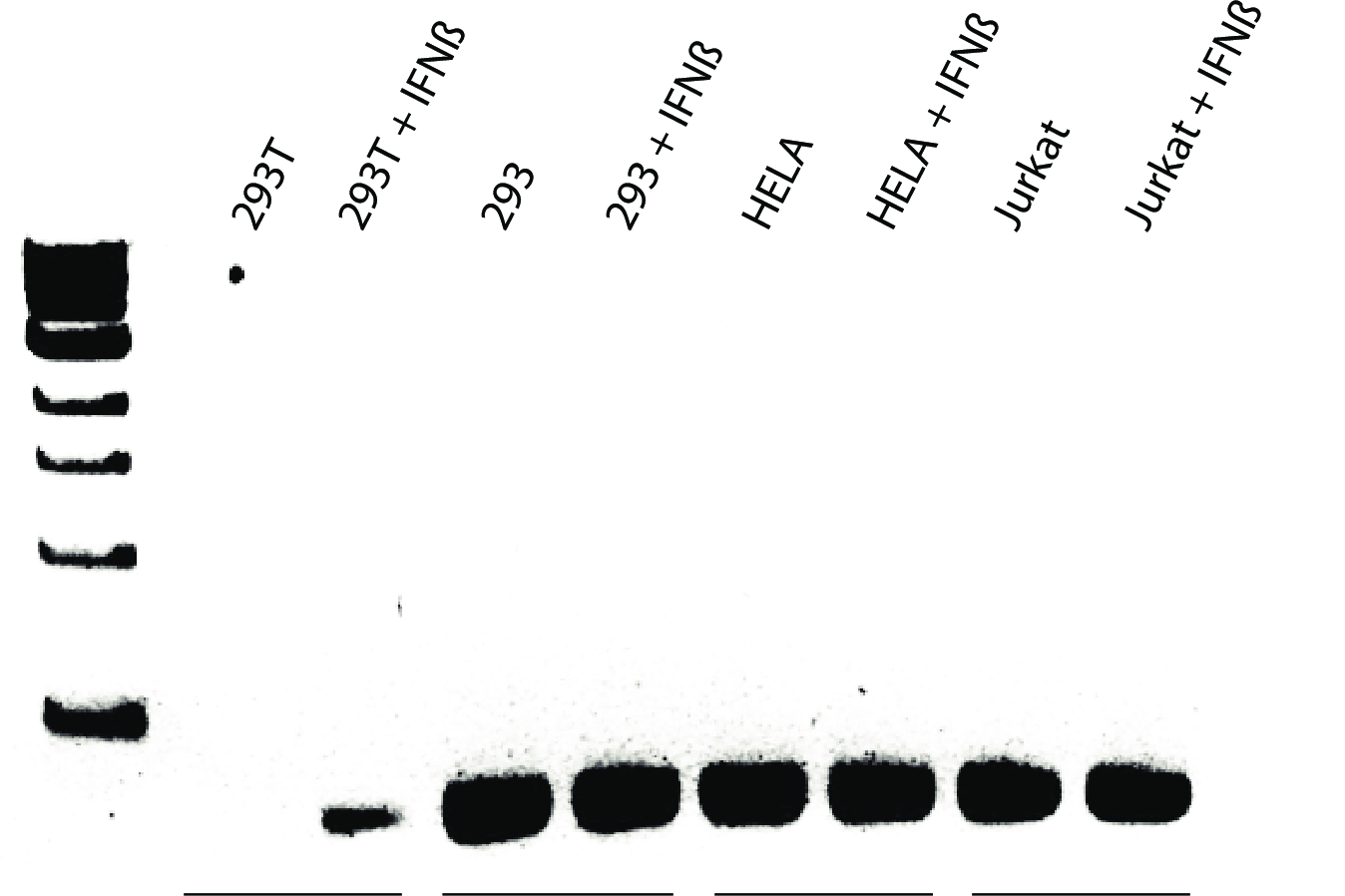

Supplement: S1 Fig — Four common human cell lines were treated with 1x10^6 IU/mL of interferon β-1b for 24 hours before cell lysates were harvested. RNA was purified from these extracts and reverse transcribed. A fragment of the SLFN11 transcript was then amplified by PCR. It can be noted that in the absence of treatment, 293T cells are hypomorphic for Schlafen11 compared to all other cells tested, as noted in [15]. (TIF) [file ppat.1006066.s002.tif]

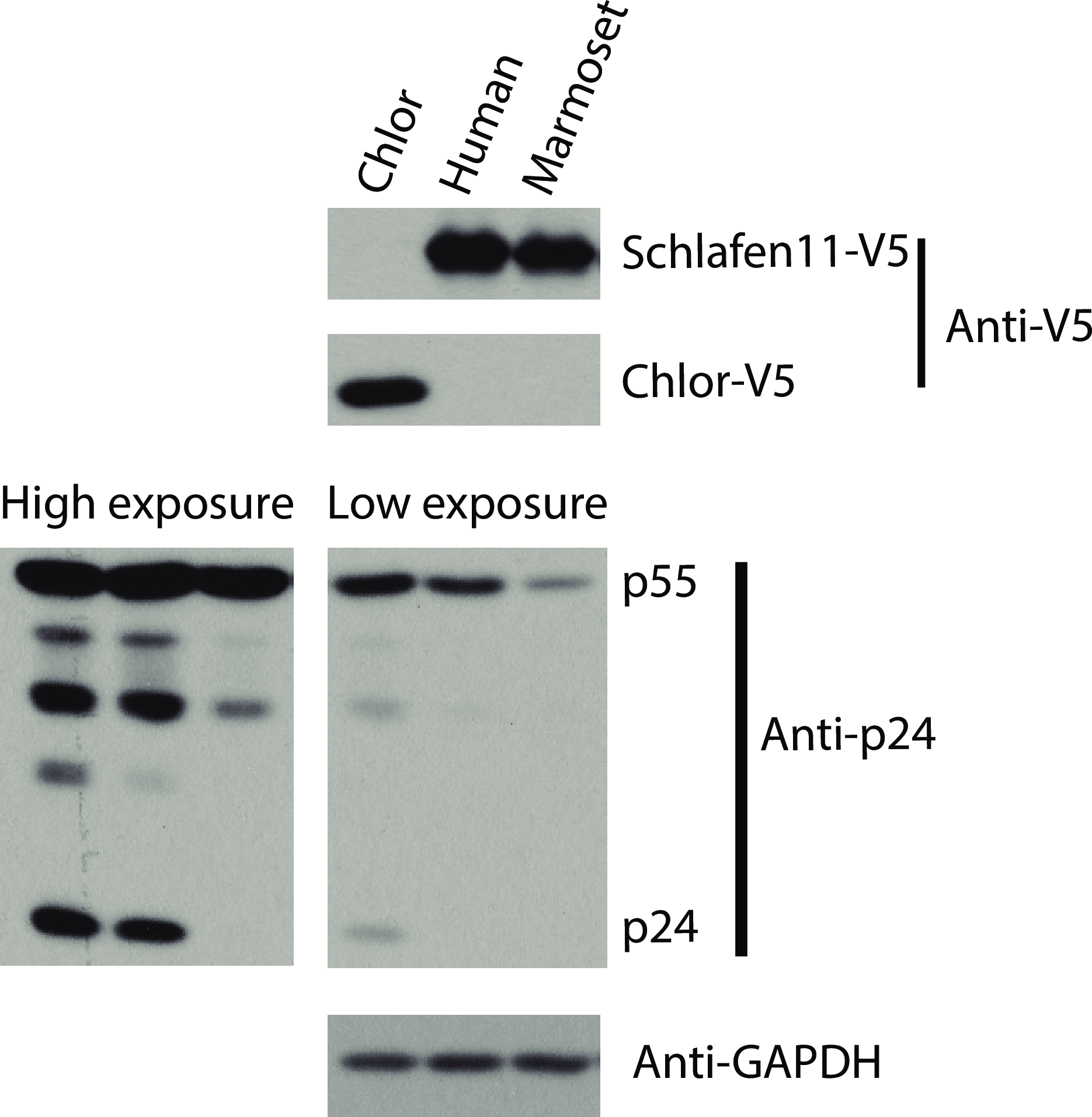

Supplement: S2 Fig — Image of a western blot showing that unprocessed Gag is affected by marmoset Schlafen11 in our experiments. (TIF) [file ppat.1006066.s003.tif]

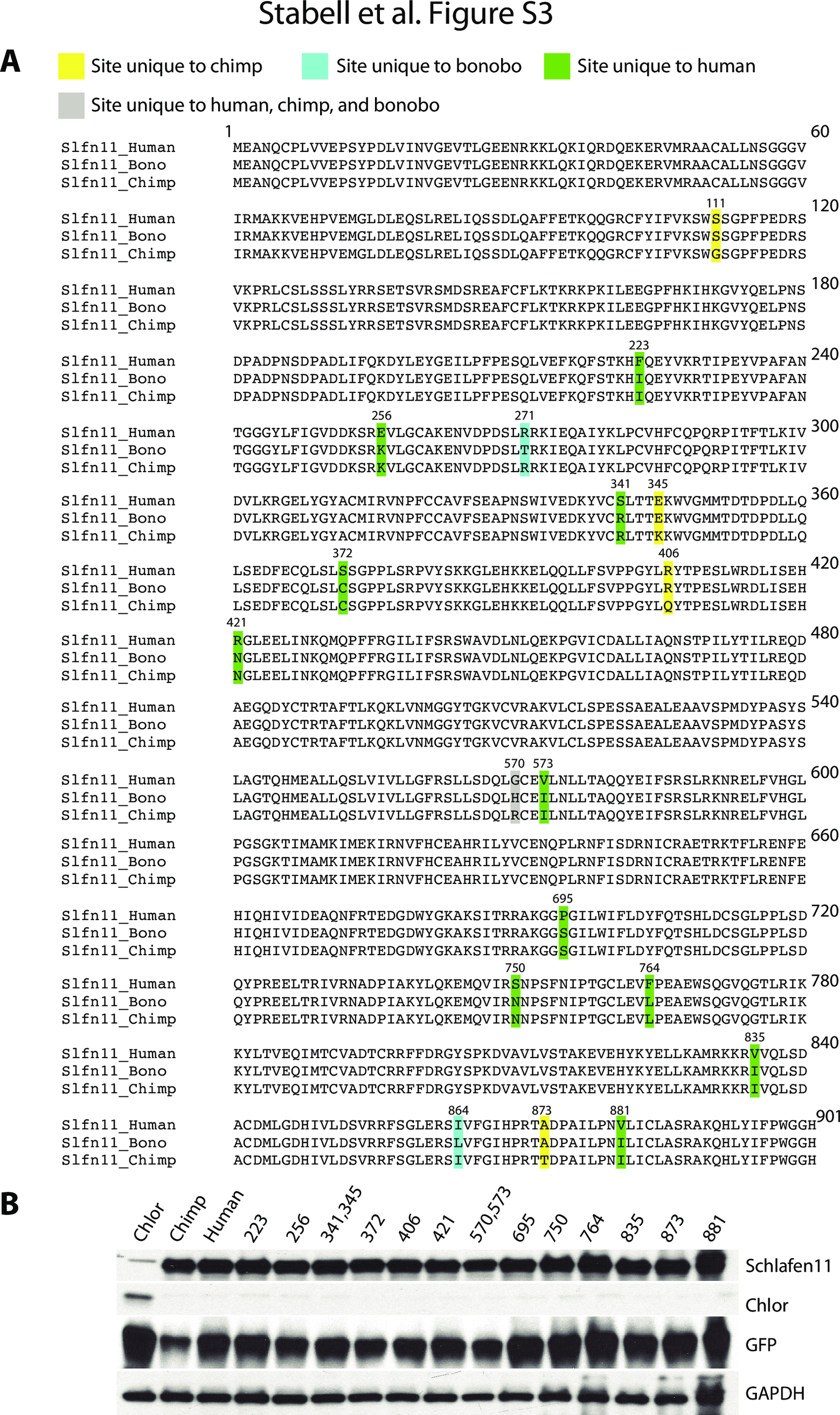

Supplement: S3 Fig — No single mutation in human Schlafen11 conveys the ability to inhibit translation (A) A multiple sequence alignment of human, bonobo, and chimpanzee Schlafen11 is shown. Differences are highlighted as indicated. (B) Site directed mutagenesis was used to change indicated residues in human Schlafen11 to those found in chimpanzee Schlafen11. Plasmids encoding these modified proteins (or chimpanzee Schlafen11 as a positive control; chloramphenichol acetyltransferase (Chlor) as a negative control) were then co-transfected into 293T cells along with a plasmid encoding GFP. Cell extracts were subject to immunoblotting and GFP protein levels were used as a read-out of translational suppression. All numbers across the top refer to positions in the human protein which were mutated to match the residue found in the chimpanzee protein. (TIF) [file ppat.1006066.s004.tif]
